# Data on multicultural education and diagnostic information profiling: Culture, learning styles and creativity

**DOI:** 10.1016/j.dib.2016.11.024

**Published:** 2016-11-18

**Authors:** Andino Maseleno, Glenn Hardaker, Noraisikin Sabani, Nabilah Suhaili

**Affiliations:** Centre for Lifelong Learning, Universiti Brunei Darussalam, Brunei Darussalam

**Keywords:** Multicultural education, Diagnostic information profiling, Culture, Learning styles, Creativity

## Abstract

This article contains data related to multicultural education and diagnostic information profiling preliminary findings. It includes the responses of 253 students. The data consists of six sections, i) culture: race, ethnicity, language and identity; ii) learning preferences: physiological and perceptual; iii) cognitive learning styles: physical, emotional and mental; iv) creativity skills and problem solving skills; v) motivation; and vi) students’ background knowledge. The data may be used as part of data analytics for specific personalized e-learning platform.

**Specifications Table**

**Table:** 

Subject area	Educational technology
More specific subject area	Personalized learning; adaptive learning technology
Type of data	Table
How data was acquired	Questionnaire
Data format	Filtered completed data
Experimental factors	There are no pre treatment samples
Experimental features	Experiments involves questionnaires that will be utilized as preliminary findings for diagnostic tool software development
Data source location	United Kingdom
Data accessibility	The data is with this article

**Value of the data**

• The data may be utilized as part of diagnostic tool software testing and development.
• The data may be utilized as a means to build learner analytics and predictive modeling software.
• The data may also utilized as a means to build adaptive portfolio for personalized learning.

## 1 Data

The data presented in this article show the responses of 253 Higher Education students completing questionnaire relating to their cultural environment, learning preferences, cognitive learning styles, and creativity. The responses may be used to seek correlation or may be selected based on subsets, identified as personalized parameters.

## 2 Experimental design, materials and methods

**Fig. 1:**
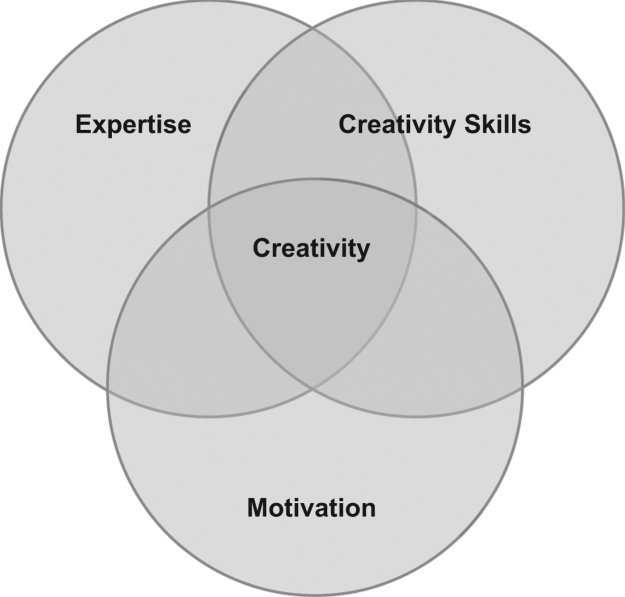
Component model of creativity (Adapted from Amabile [7]).

The research design of this study was experimental and involves questionnaires that were utilized as preliminary findings for diagnostic tool software development. The question sets consist of six sections which include culture (race, ethnicity, language and identity); learning preferences (physiological and perceptual); cognitive learning styles (physical, emotional and mental); creativity (creativity skills and problem solving skills, motivation, and subject specific knowledge). Culture has become one of the main focuses of this profiling data as it has been found to shape the way students learn [1]. This includes cultures that the students are exposed to, may it of their own and also cross culture, and is found to affect all spheres of life, including their personality and cognitions and including their own learning [1], [2], [3]. Learning preferences identify the student׳s learning preference in the context of ‘Visual’, ‘Auditory’ or ‘Kinaesthetic’ learner. The results from this section are able to integrate into Virtual Learner Management Systems to support adaptive content and associated learning. Cognitive styles is closely associated with personality traits [4]. Cognitive styles is associated with how a student organizes and processes data that has particular meaning for them [5]. It is important to analyze cognitive styles to facilitate an understanding of the various psychological and physical behaviors of an individual student [6]. The last section of the assessment concentrates on the user׳s creativity levels. Research suggests [7] that creativity will most likely occur when the three elements of expertise, task motivation and creative thinking overlap. Fig. 1 shows component model of creativity.

**Fig. 2:**
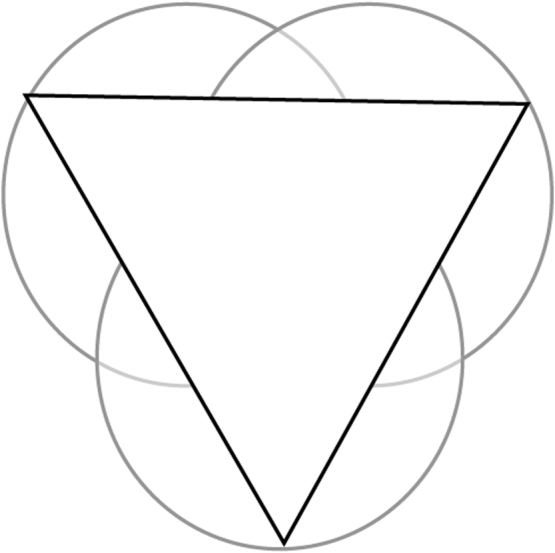
Overlay triangle.

**Fig. 3:**
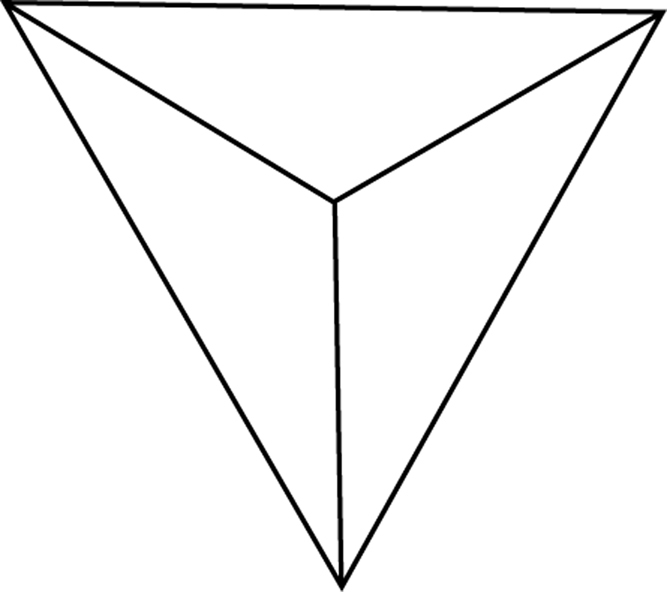
Devising scale of measurement.

**Fig. 4:**
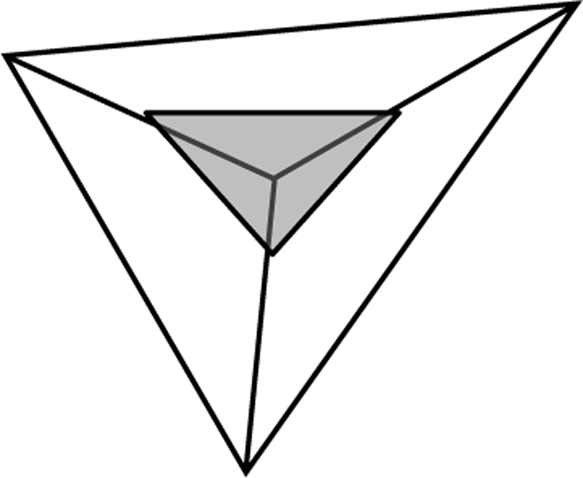
Creative user.

**Fig. 5:**
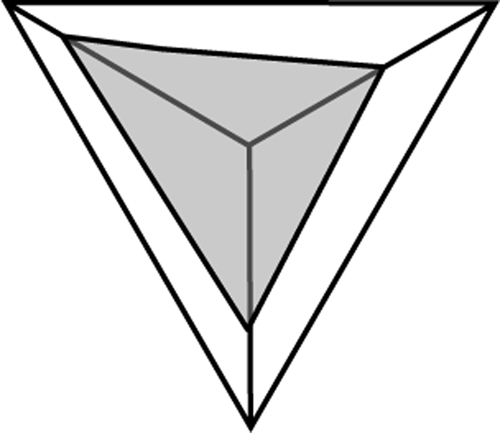
Non-creative user.

We can therefore assume that maximum creativity is achieved at the central point where the three circles overlap equally. If we place an equalteral triangle over this point so that its corners are touching the outer points of the circles (see Fig. 2), we can measure a line from each corner to the centre. If we represent the line as a “ratio” scale then we can measure and plot each factor (Fig. 3). When the 3 scores for each factor are plotted, a new triangle is drawn within the main one. As an “ideal”, a person can be considered “most creative” when their scores result in a balanced smaller equalateral triangle (Fig. 4) in the centre while a less-creative person will have an unbalanced, significantly larger acute or obtuse triangle (Fig. 5).

Applying such a theoretical perspective requires each of the parts of “understanding your creativity” to be measurable along the same “ratio” scale.
