# Supplementary material for: Data on multicultural education and diagnostic information profiling: Culture, learning styles and creativity
Source: Data Brief. 2016 Nov 18;9:1048–51. doi: 10.1016/j.dib.2016.11.024 (PMC5128012; doi:10.1016/j.dib.2016.11.024)
Supplement: Supplementary file 1 — Supplementary material [file mmc1.pdf]

Conflict of Interest

This statement is to certify that all Authors have seen and approved the manuscript being submitted. We warrant that the article is the Authors' original work. We warrant that the article has not received prior publication and is not under consideration for publication elsewhere. Conflicts of interest: None.

Yours Sincerely

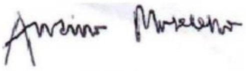

Dr. Andino Maseleno
