# Supplementary material for: Data on multicultural education and diagnostic information profiling: Culture, learning styles and creativity
Source: Data Brief. 2016 Nov 18;9:1048–51. doi: 10.1016/j.dib.2016.11.024 (PMC5128012; doi:10.1016/j.dib.2016.11.024)
Supplement: Supplementary file 2 — Supplementary material [file mmc2.zip › mmc2/Multucultural Education/Multicultural Education and Diagnostic Information Profiling.doc]

**Multicultural Education and**

**Diagnostic Information Profiling**

***Culture, Learning Styles and Creativity***

**Multicultural Education AND**

**Diagnostic Information Profiling**

**Section	Page No**

Introduction	3

1 – Culture: Race, Ethnicity, Language, and Identity…………………………………	7

2 - Learning Preferences: Physiological & Perceptual …..…………………………..	11

3 - Cognitive Learning Styles: Physical, Emotional & Mental ……………………….	16

4 - Creativity 1: Creativity Skills & Problem Solving Skills	20

5 - Creativity 2: Motivation	24

6 - Creativity 3: Subject Expertise (multiple modules)	26

Appendixes

Culture: Race, Ethnicity, Language, and Identity

Cognitive Learning Styles: Physical, Emotional & Mental

Learning Preferences: Physiological & Perceptual

Creativity in Context: Creative Thinking, Task Motivation, and Expertise

**1 - Culture: Race, Ethnicity, Language, and Identity**

This is an optional stage in the assessment. Individuals are not required to fill it in to obtain a report and the section is specifically designed to add weight to the overall profile being generated. It will be of particular use to a tutor who may be required to know more about their student.

The section directly addresses cultural diversity with a hope of better understanding the type of information and learning that will be of the most interest and relevance. There is little direction given on the style of answers required as the interpretation of the statement itself provides information on the individual’s pattern of thinking.

Today, the challenge is to create learning environments that maintain the cultural integrity of every person while enhancing their educational success. At the classroom level, culturally responsive teaching essentially involves using students’ cultural experiences and background as a medium for helping them learn important academic skills. There has also been a general agreement that the development of individual cultural identity is a major process of acceptance of the cultural norms, beliefs, attitudes and values of one cultural group rather than another. Hence multicultural education is important and necessary in order for students to be able to develop personal autonomy. To create inclusive education we need learning environments to re-configure in order to be in a proactive position to support diversity and equality.

## The scale of measurement

0123456789	10

Very Rarely

Very Frequently

Ordinal Scale

**Instructions**

Read each of the questions carefully and then use the slider provided to drag the pointer along the line to a position that you feel best represents you on the scale provided **(10 point scale as above)**

**0 = Very rarely ________________5 = Average________________10 = Very Frequently**

**culture: Race, Ethnicity, Language, and Identity**

**Questions Sets**

1. Do you feel that the environments where you learn reflect your life outside education and your general cultural background?

If this question is not appropriate to you please tick the box to skip the question

2. Do you feel cultural diversity in the classroom means it is more difficult to manage different ways of working together?

If this question is not appropriate to you please tick the box to skip the question

3. When reflecting on your behaviour do you have cultural norms, beliefs, attitudes and values that represent you as an individual?

If this question is not appropriate to you please tick the box to skip the question

4. Does your classroom environment reflect a multicultural education environment in the context of student diversity?

If this question is not appropriate to you please tick the box to skip the question

5. When reflecting on your current teachers/educators how well do they support your cultural experiences?

If this question is not appropriate to you please tick the box to skip the question

6. Do your values, attitudes and ways of thinking change depending on the people you are socialising with and the specific environment?

If this question is not appropriate to you please tick the box to skip the question

7. Do you feel knowledge is global in terms of meaning?

If this question is not appropriate to you please tick the box to skip the question

8. In your early years of education, when you were learning skills such as reading, writing and computing: was your cultural background reflected in the teaching process?

If this question is not appropriate to you please tick the box to skip the question

9. When you learn do you feel the teacher/educator proactively integrates groups from different cultural backgrounds into the learning experience?

If this question is not appropriate to you please tick the box to skip the question

10. Do you feel cultural diversity in the classroom means it is easier to work together in groups?

If this question is not appropriate to you please tick the box to skip the question

11. What level of impact have your family’s/guardians’ religious beliefs had on your current belief, norms and behaviour?

If this question is not appropriate to you please tick the box to skip the question

12. If you have any religious beliefs (passive or active), to what extent do they influence your general behaviour, beliefs, attitudes and values?

If this question is not appropriate to you please tick the box to skip the question

13. In your experience, have your teachers used examples appropriate to the diversity of the learners present in the room?

If this question is not appropriate to you please tick the box to skip the question

14. As a result of your experiences in learning, do you feel that you have an improved awareness of the cultural backgrounds of your colleagues who are of ethnic minority groups?

If this question is not appropriate to you please tick the box to skip the question

15. Do you feel cultural diversity in the classroom makes the learning process more difficult?

If this question is not appropriate to you please tick the box to skip the question

16. Do you feel that your current learning environment is enhanced through a global educational perspective?

If this question is not appropriate to you please tick the box to skip the question

17. Does the educational place where you study proactively celebrate cultural diversity and different ways of living life?

If this question is not appropriate to you please tick the box to skip the question

18. Do you feel having people with different cultural backgrounds in a learning environment makes the process more interesting and rewarding?

If this question is not appropriate to you please tick the box to skip the question

19. If your first language is different from the main language in your learning environment, are you provided with direct support?

If this question is not appropriate to you please tick the box to skip the question

20. Does the educational place where you study proactively support ethnic minority groups and people’s cultural differences?

If this question is not appropriate to you please tick the box to skip the question

21. Is bilingual support provided in the classroom/learning environment when needed?

If this question is not appropriate to you please tick the box to skip the question

22. Do your teachers challenge cultural stereotyping and overt racism?

If this question is not appropriate to you please tick the box to skip the question

**Understanding Learning Styles**

**2 - Learning Preferences: Physiological & Perceptual**

This stage addresses the user’s learning preference by determining whether he/she is a ‘Visual’, ‘Auditory’ or ‘Kinaesthetic’ learner. The results from this section will be used by a Virtual Learning Environment (VLE) to determine how information should be delivered (i.e.: as an interactive movie, a video clip, an audio clip, as text, or as a combination of each). The information may also be useful to a tutor in planning a learning session or in assessing a learner and allowing some dispensation for the difficulty the learner may experience with the style of delivery available.

Visual learners need to see, observe, record and write. They tend to absorb information best through seeing Images, Charts, Lists and Videos, etc. They will find it easiest to understand meaning through visualisation and interpretative illustration. This type of user typically interprets content best by reading, or through written description. When thinking about possibilities, the visual learner works effectively with brainstorming exercises, such as webbing and mind mapping.

Auditory learners need to talk and to listen. They will understand by listening and responding to information. For example, they will develop meaning by holding dialogue about and discussing a subject. Auditory learners typically interpret content best by listening (e.g. to a lecture) and engaging in debate. When thinking of possibilities, the auditory learner prefers to talk out ideas, interests and problems.

Tactile-kinaesthetic learners need to move, to do for themselves, to touch and be physically involved. They need structured, hands-on activities, such as creating models (e.g. a replica of a building), or designing (e.g. music or software). Kinaesthetic users gain meaning by being involved in activities. They typically need to touch and interact with what is being discussed, for example by holding and examining a model. They will typically see possibilities by immersing themselves in trial and error experimentation, such as designing and creating a solution.

## The scale of measurement

Average

Level

Very Rarely

Very Frequently

0123456789	10

Ordinal Scale

**Instructions**

Read each of the questions carefully and then use the slider provided to drag the pointer along the line to a position that you feel best represents you on the scale provided **(10 point scale as above)**

**0 = Very Rarely ________________5 = Average________________10 = Very Frequently**

**learning preference: physiological/perceptual dimension**

**Questions Sets**

1. I feel I can remember more about a subject through listening than by reading about it.

2. I generally have written directions when going somewhere new and this works better for me than if I follow verbal directions.

3. I am best able to remember important facts and ideas if I write or type them out, or if I take notes.

4. I frequently press hard with my pen while writing, or I hit the keyboard keys quite firmly when working with the computer.

5. I require supporting explanations when referring to diagrams, images or other forms of visual directions.

6. It feels very intuitive and natural to me to work with development tools, whether they are physical or digitally–based.

7. I can easily understand issues and their implications from diagrams and photographs.

8. When presented with pairs of sounds, I know when the sounds match.

9. Writing/typing help me to remember ideas and to think more clearly.

10. I use maps from the Internet to help me find my way in a new town or to find directions to a new place.

11. I feel it is easier to learn by listening to lectures, CD’s, MP3’s and file casts.

12. I play with articles such as pens, keys and other objects around me while I am learning.

13. I prefer to learn spelling and grammar by repeating the letters and phrases aloud.

14. When I check what is happening in the news, I read a newspaper or an online news source rather than listen to the radio or watch TV.

15. I seem to learn best when I am moving around and creating, repairing or improving objects.

16. I can remember things best by visualising something in my head.

17. When I was young I learnt spelling and grammar best when I wrote the words down on paper.

18. I learn more by listening to a good speaker than by reading about the same issues in a book, magazine or online.

19. I find jigsaw puzzles and other mind games are very enjoyable and engaging.

20. During learning periods I like to hold objects in my hands.

21. I prefer to listen to the news on my mobile device, radio, or online instead of reading a newspaper or magazine.

22. I often look for information on an interesting subject in books, magazines and on the Web.

23. I feel very comfortable and natural with physical contact with people (handshake, embrace).

24. I seem to follow oral directions more easily than written directions.

# **3 - Cognitive Learning Styles: Physical, Emotional & Mental**

As with learning preferences, people may also be classified by their learning style. We have categorised learners into three types: “physically orientated”, “emotionally orientated”, and “mentally orientated”. This form of categorisation is supported by European research from Riding and Cheema Cognitive Styles Analysis (CSA), and the Who list-Analytical (WA) dimension. In addition, extensive research has been conducted on learning styles by Dunn & Dunn and specifically on multiculturalism and learning styles in the Americas by Dunn & Griggs.

This section asks the user to answer a number of questions in order to assess his/her learning style.

Physically centred (P) learners learn best by observing and/or doing. They will need very limited verbal instruction but will require suitable time for both practice and completion of an activity. Ideally, the content of an activity should be practical and pertinent to the learner’s own life. Collaboration through co-operation with others can also be very effective in supporting the learning process.

Emotional/relational learners (E) operate best by listening to others and discussing the activity with others. They work most efficiently by relating the activity to their own lives and when the learning process is supported by a relaxed atmosphere. Emotional/relational learners are typically able to deal with multiple activities while learning something new. They may find reassurance in saying what they know out loud to others.

Mentally centred (M) learners absorb information by focusing on the idea or theory of the activity. They learn best when the subject concerns something they value. Typically, they are independent learners; however, they enjoy talking about ideas and viewpoints with others. Mentally centred learners concentrate deeply on specific learning tasks and find it difficult to divide attention in order to listen or watch other activities at the same time.

## The scale of measurement

Average

Level

Very Rarely

Very Frequently

0123456789	10

Ordinal scale

## Section Details

##### Instructions

Read each question carefully and then tick the answer that you feel most applies to you. Try not to think too long about the answer but go with your instinctive reaction.

**0 = Very Rarely ________________5 = Average________________10 = Very Frequently**

**Cognitive learning style: physical, emotional, mental dimensions**

**Question Sets**

1. Do you like to work on your own and take your time in your learning?

2. Do you attend specialist lectures or sessions to supplement your learning?

3. If you were given the opportunity, would you prefer one-on-one teaching sessions?

4. Do you learn best through practical examples and demonstrations?

5. Do you learn best when there is explanatory information provided to support your learning?

6. Do you learn best without prescriptive written instructions?

7. If you were given the choice, would you prefer to follow your own learning routine along your own schedule?

8. Do you prefer to follow clear guidelines when you are learning something new?

9. Do you prefer to develop your own routine without time restrictions when you learn?

10. Do you generally produce concise and useful information when you write?

11. When you write, do you generally produce many drafts before arriving at the final version?

12. Do you like to express your own opinions when you write?

12a. When you are learning something new, do you like to relate the new topic to something you already know?

13. When you are learning something new, do you usually begin by forming a visual image of the topic?

14. When you are learning something new, do you like to begin by gathering as much information as possible?

15. Do you feel that beginning a new topic is a relatively easy process?

16. Do you usually focus on the practical details of what you have learnt?

17. Is the work you are given by teachers usually pretty easy for you?

18. Do you feel it is difficult to begin learning something new?

19. Do you find it difficult to focus on the details and put what you learn into practice?

20. Is it difficult for you to complete tasks when learning?

21. Do you usually begin new activities or tasks by reading instructions and then considering the learning materials at hand?

22. Do you usually begin new activities or tasks by referring to the learning materials at hand and then reading instructions?

23. Do you usually begin new activities or tasks by looking at the learning materials and then developing your own way of completing the task?

24. Do you usually relate a new activity or task to the skills you need to learn in order to complete the task?

25. Do you usually begin a new learning process by relating it to other things you already know?

26. Do you usually begin a new learning process by simply getting on with the activity?

27. If you were asked to learn something technical, would you prefer to begin by experimenting?

28. If you were asked to learn something technical, would you prefer to begin with verbal instructions from a teacher?

29. If you were asked to learn something technical, would you prefer to begin by reading instructions?

30. Do you prefer to learn new ideas and theories by discussing them with colleagues?

31. Do you prefer to learn new ideas and theories by associating them with real-world applications?

32. Do you prefer to learn new ideas and theories by reading about them?

33. Do you feel it is most important to be able to apply what you have learnt to something in real life?

34. Do you feel it is most important to be able to demonstrate or explain what you have learnt to your colleagues and friends?

35. Do you feel it is most important to be able to understand the core concepts of the subject you have learnt?

**Creativity in Context:**

**Creative Thinking, Task Motivation & Expertise**

The last stage of the assessment concentrates on the user’s creativity levels. The stage comprises 4 parts that examine 3 central elements of our personal creativity. For the purposes of this experiment it is accepted that creativity is achieved in a subject or activity when the right mixture of creativity and problem solving skills, motivation and expertise are combined. This is based on the “Componential Theory of Individual Creativity” which assumes that “all humans with normal capacities are able to produce at least moderately creative work in some domain, some of the time – and that social environment (the work environment) can influence both the level and frequency of creative behaviour.” (Amabile, 1997)

Research suggests that creativity will most likely occur when the three elements of Expertise, Task Motivation and Creative Thinking overlap as demonstrated in the diagram below.

**Expertise**

**Creativity Skills**

**Motivation**

**Creativity**

Fig 7.0 Component Model of Creativity (Adapted from Amabile, 1997)

We can therefore assume that maximum creativity is achieved at the central point where the three circles overlap equally. If we place an equalteral triangle over this point so that its corners are touching the outer points of the circles (see Fig 8.0), we can measure a line from each corner to the centre. If we represent the line as a “ratio” scale where 0 is at the triangle’s corner and 10 is at its centre, then we have a measurement scale along which to plot each factor (Fig 9.0). When the 3 scores for each factor are plotted, a new triangle is drawn within the main one. As an “ideal”, a person can be considered “most creative” when his/her scores result in a balanced smaller equalateral triangle (Fig 10.0) in the centre while a less-creative person will have an unbalanced, significantly larger acute or obtuse triangle (Fig 11.0)

Fig 10.0 Creative user

**Creativity**

Fig 11.0 Non-creative user

Fig 9.0 Devising scale of measurement

Fig 8.0 Overlay triangle

Applying such a theoretical perspective requires each of the parts of “Understanding your creativity” to be measurable along the same “ratio” scale.

**4- Creativity 1: Creativity & problem solving skills**

The first part of this section assesses both the user’s ability to behave creatively and the creative tools to which he/she has access. By understanding these assets it is possible to understand how the user is likely to employ the knowledge he/she gains from the learning environment; in this case, whether he/she will be creative in the field of Internet marketing and E-commerce.

Research suggests that users who have high creativity skill levels will also learn in a creative way, and are therefore likely to expand the individual knowledge, understanding and skills base that they obtain from the course. Users with lower creativity skill levels will require some introduction to creative thinking skills, problem solving strategies and help to make best use of the core information obtained from the knowledge components.

## The scale of measurement

Average

Level

Very rarely

Very Frequently

0123456789	10

Ordinal scale

In theory, it should be impossible to assign a frequency of 0 or 10. Most people are likely to position themselves as average or below (natural levels of creativity); those who have adopted some form of problem solving technique or creative style are more likely to position themselves as above average for some questions. The vast majority of people have not been taught any of these specialised kinds of skills; it will be necessary to include standard knowledge components that are not subject specific and which address purely the improvement of creativity skills. Those who have made creative achievements in the past will likely position themselves higher on the scale than those who have not.

***Very Frequently:*** *Users whose answers result in a perfect 10 are able to employ an absolute wealth of creativity and problem solving skills. They have a huge history of creative achievements and situations where they have effortlessly solved problems or generated ideas to the maximum benefit of all concerned. Regarded as geniuses in their field, they are likely to be at the top of their profession and have had formal training in thinking creatively.*

***Average Level:*** *The average level will be the closest indicator for most users. Users who register in the middle of the scale will be able employ creativity skills that allow them to solve general problems, yet they still have to struggle to solve more complex problems. With prompting and guidance some complex problems can be solved, but it is still easy to make mistakes. The user is not exceedingly creative on his/her own, but in groups or with coaching some good results can be achieved. They are confident carrying out basic tasks but are likely to be less certain with more advanced work and will undoubtedly require training.*

***Very Rarely:*** *A user totally devoid of creativity skills will score a perfect 0. Such a candidate will struggle daily even with the simplest problems or decisions and is likely to have a below-average level of general intelligence. Such a person is, at best, a complete novice in his/her field and is likely to have no understanding of the general subject area. This user has no concept of even the simplest and most widely-used creativity skills.*

##### **Instructions**

Read each of the questions carefully and then answer using the scale provided. Try not to think too long about the answer but go with your instinctive reaction.

**0 = Very Rarely________________5 = Average________________10 = Very Frequently**

1. How often do you consider yourself to be creative?

2. When you have a task to complete do you think to yourself I will do it now?

3. How often do you employ methods and approaches to working/learning that enable you to organise your time effectively?

4. Do you feel confident when communicating new ideas to your regular work colleagues?

5. When you have an original idea, do you immediately consider problems of practical usage and application?

6. Do you feel that you immediately do activities that need doing?

7. Are you able to plan your time to alternate between different activities such as work and mental relaxation?

8 When you can not find a solution to a problem, do you continue searching for other alternatives?

9 Do you feel that you work well independently?

10 Do you feel you are a person who takes (reasonable) risks?

11 Do you feel comfortable making (seemingly) ridiculous suggestions?

12 Do you feel that you are persistent when you are faced with a difficulty or a challenging situation?

13 Do you feel you are flexible in how you deal with situations and problems?

14? Do you follow your own way of dealing with challenges even though this may be risky?

15 Do you accept instability and discontinuity in your work?

16 Do you do tasks because you feel you want to do it?

17 When you are thinking about an idea or subject, do you tend to search for themes, concepts and principles to understand the bigger picture?

18 Do you feel you have a high level of self-discipline in your work?

19 Do you challenge or question established procedures and methods of doing something?

20 Do you develop new ways of undertaking tasks and activities?

21 If you are unclear if a work situation is right or wrong would you feel comfortable with this situation?

22 Would you find it easy to identify 50 unusual uses for an object in ten minutes?

23 Do you feel you are habit-forming in your actions?

24 Do you feel uncomfortable about being different or “out of tune” with others?

25 When you are introduced to a new activity, do you use techniques and strategies to overcome problems?

26 Do you consider yourself to be perceptive in your behaviour?

27 How often do you complete a task even though you do not feel you are in an appropriate “state of mind”?

28 Do you feel you have an active imagination when doing new and/or regular activities?

29 Do you feel you are generally a confident person?

30 How frequently have produced original ideas in the past?

## Scoring Procedure

To obtain the user’s final mark, an average is taken from all the answers provided. This is then plotted on the scale as explained above.

## 5 - Creativity 2: motivation

The following part of the assessment is designed to assess the user’s relevant motivational skills. The user’s motivation level will be key to understanding how creative they are likely to be when applying the knowledge they will acquire from the training.

Users who have high motivational levels are likely to be better learners when it comes to using the knowledge components. Those with a low motivational score are less likely to be interested. This assessment has a number of comparisons with the previous section on cognitive learning styles. There may be opportunity to combine or re-use the results from that section to enhance the findings here.

## The scale of measurement

0123456789	10

Very Rarely

Average

Very Frequently

Unlike the Creativity scale, it is more plausible that a user may rate his/herself as a 0 or a 10 on this scale, although this is still highly unlikely. A user who rates him/herself as a perfect 0 will not benefit from the course and is indicating that he/she has no interest whatsoever in a career. It is possible but still very unlikely that a user would rate him/herself as a 10. Such a user could be an entrepreneur running a company and who is entirely focussed on a professional career.

***Very Frequently:*** *Complete and utter enthusiasm for every aspect of the job. Requires no external motivation and is never de-motivated by external sources. Work does not feel like work because the career would be an interest, hobby or pastime anyway.*

***Average Motivation:*** *The user whose average falls at the perfect mid-point on the scale finds half the work to be of interest, an inspiration for success and achievement. The other half of the work is menial, monotonous and uninteresting. The two sides often cancel out the candidate’s motivation, leaving him/her indifferent about the work. Improving intrinsic motivation is not impossible but high amounts of extrinsic motivation can be very helpful.*

***Very Rarely:*** *Every aspect of the work and environment is so de-motivating that even if presented with high wages and a benefits package, there is still nothing to inspire the candidate. The very thought of work is enough to bring on immediate depression and loathing. The line of work is anti everything the person wanted from a career and the environment is completely non-conducive to success and happiness.*

A user who is keen to succeed in his/her role and/or is excited about the prospect of expanding knowledge should achieve a rating between 6 and 9. A user who is either de-motivated at work and/or is sceptical about it should expect a rating between 1 and 4. However, results are dependent on personality aspects, which may skew the expected outcomes.

**Section Details**

##### Instructions

Read each of the questions carefully and then answer using the scale provided. Try not to think too long about the answer but go with your instinctive reaction.

**0 = Very Rarely ________________5 = Average________________10 = Very Frequently**

1 Do you feel that the people you learn with on a regular basis are interesting?

2 Do you consider the learning you undertake on a regular basis to be a satisfying experience?

3 How often is there clear communications between you and the people you regularly learn with?

4 How often are your highest achievements in the learning environment recognised and rewarded?

5 How often do you feel the deadlines that your supervisor/tutor sets for you are realistic?

6 Can the people you learn with effectively manage failure in the course of their daily activities?

7 Are the people you regularly learn with outstanding at the learning activities assigned?

8 Do you feel your work pushes you to undertake activities that are achievable but challenging?

9 When you are learning in a group, are your colleagues helpful and respectful in the collaborative activities?

10 How often can you set yourself reasonably challenging goals in your learning and get feedback on your results?

11 Do you feel that most of the work/learning you undertake relates to your own personal interests?

12 Do you feel there is a sufficient amount of natural light in the space where you learn?

13 How often do you feel you become absorbed in the activities of learning?

14 Is the place where you learn a friendly environment?

15 Do you feel that your family or close friends are supportive of your learning development?

16 Are the people you learn and collaborate with on a regular basis competent at what they do?

17 How often do you expect to receive constructive criticism for your work?

18 How often do you feel your learning activities are appropriately monitored and evaluated?

19 Are you given sufficient resources to adequately undertake you learning activities?

20 How often are you in cooperation with the other people you work with?

21 How often are you encouraged by your colleagues in your efforts to be innovative in your learning?

22 Do you feel that the people you learn and interact with on a regular basis are cooperative?

23 Do you consider your achievements in learning important?

24 Is the place where you learn effectively managed?

25 Would you describe your learning experiences as challenging?

26 Are your colleagues constructive when they comment on and challenge your ideas?

27 How often do you feel you have influence over your learning activities in terms of making your own decisions and choices in direction?

28 Do you feel that learning has been a rewarding experience for you?

29 Do you feel your general activities are valuable to others?

30 How often do you feel that your learning is enabled through flexible procedures?

**6 - Creativity 3: subject expertise (Compulsory Module from options)**

The section is designed to assess the relevant level of knowledge the user has in a certain field; in this case the main topic is Internet Marketing and E-commerce. The “Expertise” part of the 4-fold assessment will probably have the most importance as it will determine what level of information with which the user will best be able to cope.

It is important to consider the range of possible users who may be answering the questions. It will be necessary to assess at an early stage whether a user is better suited to the basic elements of training or the more advanced. If this assessment is not made, the user may either feel as if he/she is wasting time or being patronised.

## The scale of measurement

0123456789	10

Complete

Novice

Average

User

Total

Expert

In theory it should be impossible for a user to be assigned a rating of 0 or 10. A user who achieves a 10 has no need for the course and a user who scores a zero is unlikely to even understand what the context of the course is.

***Total Expert:*** *The user rated at this level would already be at a point where he/she could learn nothing new about the Internet, E-commerce, and the most advanced principles of e-marketing.*

***Average Users:*** *Would typically have regular access to a computer and the Internet, although this would not be of any major importance to their daily routine and would not count as one of their main leisure activities. They will understand most of the basic principles of how the Internet works but have no immediate need to understand more in-depth workings. Similarly, their main experience of e-marketing will have been passive rather than active and they will have a rudimentary idea of how the principles of everyday marketing can be transferred to the web.*

***Complete Novice:*** *This unlikely user would have no concept of the existence of the Internet, E-commerce, computers or the most basic principles of marketing.*

**QUESTION SET (SAMPLER ONLY)**

**Instructions:** When adding or modifying a question set, it is extremely important to fill in every field correctly; a mistake here will cause an inaccurate result when assessing learners. It may be appropriate to prepare and test the questions on paper before entering them in the system.

NOTE: All fields marked with # are compulsory.

Top of Form

| **Name of set:** | # |
| --- | --- |
| **Authors name:** | # |
| **Scale option 1:** | # |
| **Scale option (mid-point):** |  |
| **Scale option 2:** | # |
| ** Ensure that your scale extremities (Option 1 / 2) are absolutes, i.e. Never / Not At All or Totally / Constantly. For instance, if the learner is asked to "Use the scale provided to rate how often you watch television", the possible answers should equate to a scale between two virtually impossible extremities: (0) would correspond to a user who does not even know what a television is and has never seen one; (10) would correspond to a user who watches television 24 hours a day, non-stop.* | |
| Subject Area #1 \| \|; Ques. No. \| Ques. Text \| Subject Area #2 \| \|; Ques. No. \| Ques. Text \| Subject Area #3 \| \|; Ques. No. \| Ques. Text \| Subject Area #4 \| \|; Ques. No. \| Ques. Text \| Subject Area #5 \| \|; Ques. No. \| Ques. Text \| Subject Area #6 \| \|; Ques. No. \| Ques. Text \| Subject Area #7 \| \|; Ques. No. \| Ques. Text \| Subject Area #8 \| \|; Ques. No. \| Ques. Text \| Subject Area #9 \| \|; Ques. No. \| Ques. Text \| Subject Area #10 \| \|; Ques. No. \| Ques. Text \| | |
